# Effects of electrical stimulation with alternating fields on the osseointegration of titanium implants in the rabbit tibia - a pilot study

**DOI:** 10.3389/fbioe.2024.1395715

**Published:** 2024-07-24

**Authors:** A. Klinder, F. Möws, J. Ziebart, Y. Su, C. Gabler, A. Jonitz-Heincke, U. van Rienen, M. Ellenrieder, R. Bader

**Affiliations:** ^1^ Research Laboratory for Biomechanics and Implant Technology, Department of Orthopedics, Rostock University Medical Center, Rostock, Germany; ^2^ Institute of General Electrical Engineering, University of Rostock, Rostock, Germany; ^3^ Department of Ageing of Individuals and Society, Interdisciplinary Faculty, University of Rostock, Rostock, Germany; ^4^ Department of Life, Light and Matter, Interdisciplinary Faculty, University of Rostock, Rostock, Germany; ^5^ Department of Orthopedics, Rostock University Medical Center, Rostock, Germany

**Keywords:** direct electrical stimulation, alternating fields, osseointegration, implant, animal study

## Abstract

**Introduction:** Electrical stimulation has been used as a promising approach in bone repair for several decades. However, the therapeutic use is hampered by inconsistent results due to a lack of standardized application protocols. Recently, electrical stimulation has been considered for the improvement of the osseointegration of dental and endoprosthetic implants.

**Methods:** In a pilot study, the suitability of a specifically developed device for electrical stimulation *in situ* was assessed. Here, the impact of alternating electric fields on implant osseointegration was tested in a gap model using New Zealand White Rabbits. Stimulation parameters were transmitted to the device via a radio transceiver, thus allowing for real-time monitoring and, if required, variations of stimulation parameters. The effect of electrical stimulation on implant osseointegration was quantified by the bone-implant contact (BIC) assessed by histomorphometric (2D) and µCT (3D) analysis.

**Results:** Direct stimulation with an alternating electric potential of 150 mV and 20 Hz for three times a day (45 min per unit) resulted in improved osseointegration of the triangular titanium implants in the tibiae of the rabbits. The ratio of bone area in histomorphometry (2D analysis) and bone volume (3D analysis) around the implant were significantly increased after stimulation compared to the untreated controls at sacrifice 84 days after implantation.

**Conclusion:** The developed experimental design of an electrical stimulation system, which was directly located in the defect zone of rabbit tibiae, provided feedback regarding the integrity of the stimulation device throughout an experiment and would allow variations in the stimulation parameters in future studies. Within this study, electrical stimulation resulted in enhanced implant osseointegration. However, direct electrical stimulation of bone tissue requires the definition of dose-response curves and optimal duration of treatment, which should be the subject of subsequent studies.

## 1 Introduction

The piezoelectricity of bone and the effects of electric currents on osteogenesis were described almost 70 years ago (Fukada and Yasuda, 1957; Bassett et al., 1964; Bassett, 1968; Fukada, 1968). Due to its effects on bone remodeling, electrical stimulation became a promising approach in bone repair and attenuating bone loss in orthopedic and trauma surgery (Ehnert et al., 2019; Ganse, 2024). Considerable research was conducted to investigate the ability of electrical signals to affect the regenerative behavior of bone cells (Devet et al., 2021). However, the therapeutic application of the regenerative potential of electrical stimulation is still limited despite the overwhelming evidence from *in vitro* and *in vivo* studies (Bhavsar et al., 2020). So far, electrical stimulation is mainly used as a last resort in fracture healing in delayed repair or non-unions (Griffin and Bayat, 2011; Griffin et al., 2011). Clinicians stated that, apart from high costs, the reasons for the limited usage of electrical stimulation were impractical and difficult-to-use devices that produced inconsistent results and were prone to complications (Bhavsar et al., 2020). Crucial patient compliance in external stimulation is hampered by skin irritation in capacitive coupling (CC) stimulation or the weight of the devices in pulsed electromagnetic field (PEMF) stimulation. For direct internal stimulation the main disadvantages are the two necessary surgeries required to implant and remove the stimulation device. Additionally, the use of batteries and their possible dislocation represents a further problem.

In recent years, new concepts have been developed to make treatment with electrical stimulation more accessible. In particular, combining bone tissue engineering and electrical stimulation seems promising (Leppik et al., 2020; Hao et al., 2021). Leppik et al. used electrical stimulation to steer mesenchymal stem cells (MSCs), which were seeded onto bridging scaffolds for large bone defects, into osteogenic differentiation (Leppik et al., 2018). Contrarily, when MSCs, that were pre-differentiated by electrical stimulation *ex vivo*, were applied to femur defects in Sprague–Dawley rats, there were no advantages, thus suggesting that local electrical stimulation is required for the beneficial effects on bone healing (Bianconi et al., 2023). An *in vitro* study also showed that the concentration of intracellular calcium ions was only enhanced by direct electrical stimulation, but not by electrochemically conditioned medium (Bielfeldt et al., 2023). This indicates that local electrical stimulation is necessary to continue during the entire healing process. Another approach to achieve this is to directly utilize the conductive properties of scaffolds in large bone defects (Careta et al., 2023; Dixon et al., 2024). Similarly, bone cement with piezoelectric biological activity was tested regarding its ability to promote bone regeneration and osseointegration (Wang et al., 2024). The mechanisms of osseointegration are similar to those in fracture healing. While in fracture healing the improvement of bone regeneration with electrical stimulation might be sufficient, certain conditions such as tooth loss, amputation of limbs, but also osteoarthritis in the major joints still require the implantation of permanent prostheses. Electrical stimulation is thought to enhance the osseointegration of those metallic implants (Ehrensberger et al., 2020; Faoussi et al., 2024). The survival of permanent implants depends substantially on their successful integration into the surrounding bone and the formation of new bone tissue on the surface of the implant to guarantee a stable anchorage (Xiao et al., 2022). While the implant material represents an important factor for treatment success (Albrektsson et al., 1983), the underlying biological events, which comprise the three main stages of the formation of new bone (up to day 28), bone mass adaptation, and bone structure adaptation, are similar to fracture healing (Pandey et al., 2022). The cascade of events, which is activated by trauma to the bone, unfolds with the coating of the implant by the proteins from the surroundings. This protein layer facilitates the adhesion, migration and differentiation of bone progenitor cells. Different from fracture healing, in implant osseointegration, the entire population of progenitors differentiates into osteoblasts with subsequent intramembranous ossification (Pandey et al., 2022). The fact that alternating electric fields enhance the differentiation processes in osteoblasts, including biomineralization, was already shown *in vitro* (Sahm et al., 2022).

Thus, the effects of electrical stimulation on bone regeneration may also be beneficial in the osseointegration of dental or endoprosthetic implants. First approaches were reported, mainly from oral implantology with the testing of dental implants in animal models (Pettersen et al., 2022). The use of bioelectricity is a common principle in dental medicine (Min et al., 2024). However, the concept is also of interest in orthopedics to improve the anchorage of uncemented endoprosthetic implants (Zimmermann et al., 2021). Especially in arthroplasty patients who suffer from comorbidities that impair the osseointegration of implants such as osteoporosis, diabetes or rheumatoid arthritis (Aro et al., 2012; van Hamersveld et al., 2021; D’Ambrosio et al., 2023) the improvement of the interfacial bone integration might prevent implant loosening. As an additional benefit, the combination of joint arthroplasty and electrical stimulation circumvents some of the disadvantages associated with internal stimulation (Nicksic et al., 2022). Due to implantation of a joint endoprosthesis, a second surgery is not required since the implant, including a built-in electrical stimulation device, can remain permanently in the human body. Thus, there are no additional surgery-associated risks for the patient due to the therapeutic use of electrical stimulation.

In a pilot study, the suitability of a specifically developed electrical-stimulating device was assessed in an animal model that has been widely used to evaluate osseointegration (Götz et al., 2004). The impact of alternating electric fields on implant osseointegration was tested in a gap model using New Zealand White Rabbits. Stimulation parameters were transmitted to the device via a radio transceiver, thus allowing for real-time monitoring and, if required, variations of stimulation parameters. The effect of electrical stimulation on implant osseointegration was quantified by the bone-implant contact (BIC) assessed by histomorphometric (2D) and µCT (3D) analysis.

## 2 Materials and methods

### 2.1 Implant design and stimulation parameters

**FIGURE 1:**
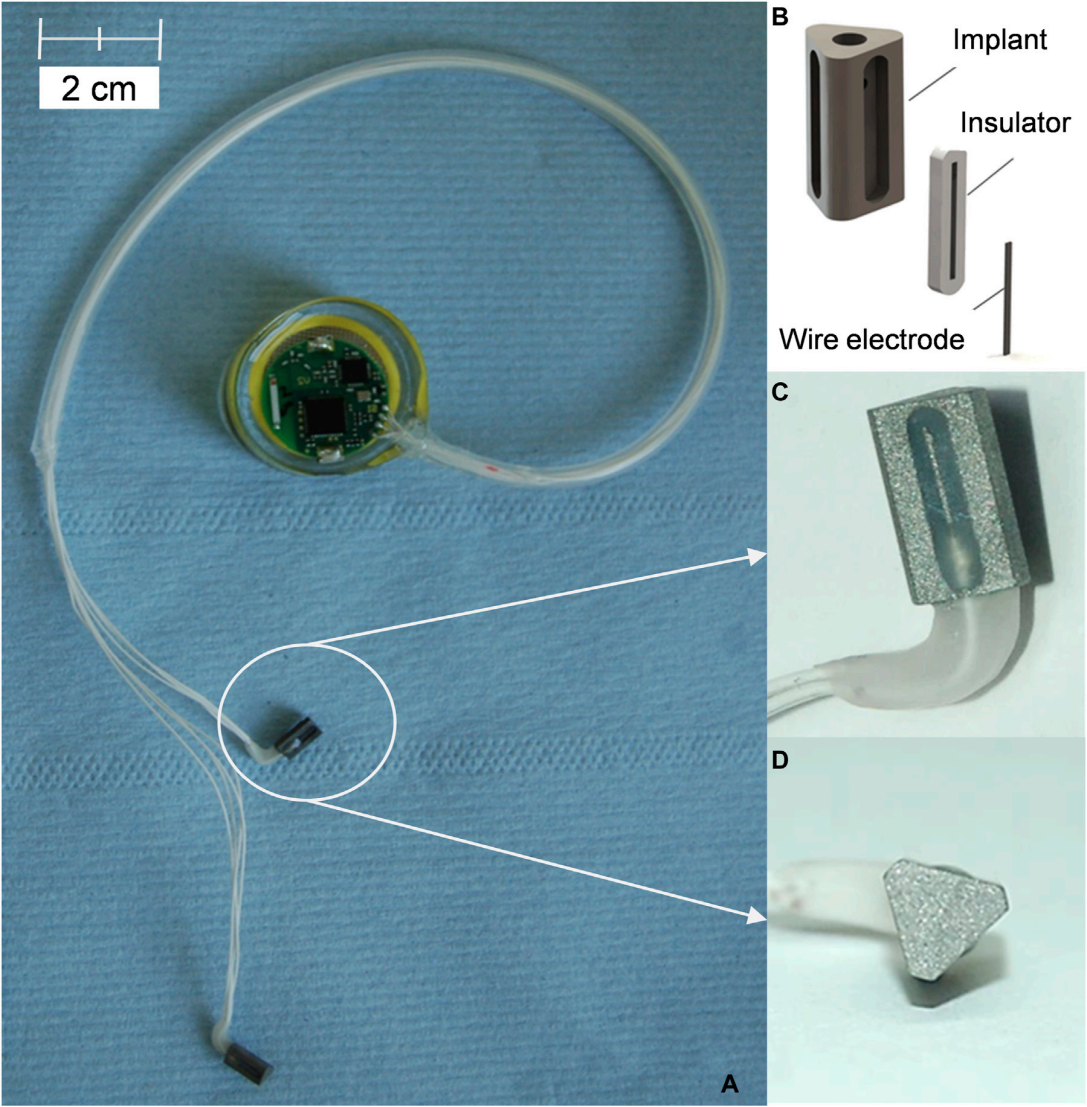
Implants and stimulation device. **(A)** Assembled stimulation device connected to implants prior to implantation. **(B)** Schematic presentation of the implant design. **(C)** Lateral view of the implant. **(D)** Triangular base of the implant.

The custom-made electrical stimulation system comprised an implant, the connecting cables, and a miniature stimulation device including a 3 V battery (Figure 1). The implant resembled a prism of 7.0 mm in length with a round-corner, triangular base of 4.4 mm diameter and was manufactured from Ti6Al4V alloy by primec GmbH (Bentwisch, Germany). An implant with a triangular base was chosen to create a gap between the bone (round drill hole) and the implant’s outer surface, which was supposed to be bridged by the newly synthesized bone, thus creating a defect model. The titanium implants had rough surfaces (surface roughness R_z_ = 12.9 ± 1.6 µm) to facilitate better adherence of osteoblasts. Additionally, the implant contained a central borehole to insert the connecting cables (copper wires coated with fluorinated ethylene propylene as insulating material) and three lateral boreholes that allowed exposure to the electrodes (Ti6Al4V wire). Each implant was connected to the miniature stimulation device via four cables – three wires carrying an electric current (electrodes) and one ground wire. For most of the length of the connection, the four cables were sheathed in a silicon tube, while the entry point of the cables into the implant was additionally reinforced with a more durable silicon (MasterSil 151Med, Master Bond Inc., Hackensack, NJ, United States). Figure 1 shows an illustration of the implant system.

The developed miniature electrical stimulation device was manufactured by Dorazil GmbH (Berlin, Germany) and contained a 3 V lithium cell as an energy source. The design of the stimulation device was described previously by Plocksties et al. (2020). Briefly, the circular-shaped printed circuit board contained an off-the-shelf MSP430F2274 microcontroller, a 2.4 GHz RF interface based on a TI CC2550 transceiver, and an output unit that approximated an alternating current (AC) sine voltage. With a diameter of 25.4 mm, a height of 1.93 mm, and a weight of 0.7 g at a total volume of 977 mm^3^, the stimulation device was suitably small for animal studies. The entire device, including the electronics on the circuit board, was encapsulated in a biocompatible polymer (NuSil™ MED1-4213 and MED3-4213, Avantor, VWR, Deutschland). The entry point of the cables into the miniature stimulation device was additionally sealed by several cycles of embedding it in silicone (MasterSil 151Med, Master Bond Inc., Hackensack, NJ, United States). Each stimulation device was able to cater for two implants.

The stimulation parameters were transmitted to the stimulation device over a radio-frequency (RF) interface, enabling a wide range of adjustable parameters. At the same time, feedback control allowed monitoring of whether the stimulation device was still working correctly.

### 2.2 Numerical simulation of implant properties in the rabbit knee

Before animal testing, numerical simulation was used to evaluate the electric field distribution in the animal bone and on the implant’s surface of the implant. The procedure was carried out according to the previously published, detailed protocol to simulate electrical stimulation in rabbit bone (Su et al., 2016). Briefly, the bone model was created in COMSOL (COMSOL Multiphysics® version 4.3b, Comsol AG, Göttingen, Germany) and was integrated into iSIGHT to run the optimization. The modeling in COMSOL was launched through MATLAB batch mode. Frequency and signal waveform were kept constant at 20 Hz and sinus wave. Both the cancellous and cortical bone in the rabbit knee were considered homogenous and isotropic to reduce the complexity of the calculation. Dielectric properties of the rabbit bone, i.e., the initial electric conductivity and permittivity of the tissues, were used as reported by Su et al. (2016). The material properties of the designed implants in the numerical simulation were incorporated into the model based on data sheets from the manufacturer. Table 1 shows the dielectric properties of the adjacent tissues and the implant material used in the electrical simulation at 20 Hz.

**TABLE 1:** Dielectric properties (electric conductivity σ and relative permittivity ε_γ_) of the implant material and adjacent tissues.

Tissue/material	Electric conductivity σ (S/m)	Relative permittivity ε_r_
Ti6A14V	7.407 × 10^5^	1
Blood	0.7	2,560
Cancellous bone	0.078902	4,020,200

A parametric study was carried out to ensure that the majority of the implant surface exhibited the optimum electric field interval of 5–70 V/m. For the parametric study, all parameters were kept constant except for the peak voltage that was varied from 50 mV to 300 mV in 50 mV steps.

### 2.3 Animal testing

The animal experiments were approved by the local review board of the Landesamt fuer Landwirtschaft, Lebensmittelsicherheit und Fischerei M-V (LALLF MV, reference number AZ LALLF M-V/TSD/7221.3-1.1-076/12). The animal study complied with policies and principles established by the Animal Welfare Act and the NIH Guide for the Care and Use of Laboratory Animals. For this purpose, nine female New Zealand White rabbits (Envigo RMS GmbH, Hillcrest, United Kingdom) with an average weight of 3.66 ± 0.30 kg and an age of 6 months were used in this pilot study. New Zealand White rabbits are a well-established experimental model for preliminary research regarding the osseointegration of titanium implants (Götz et al., 2004).

**FIGURE 2:**
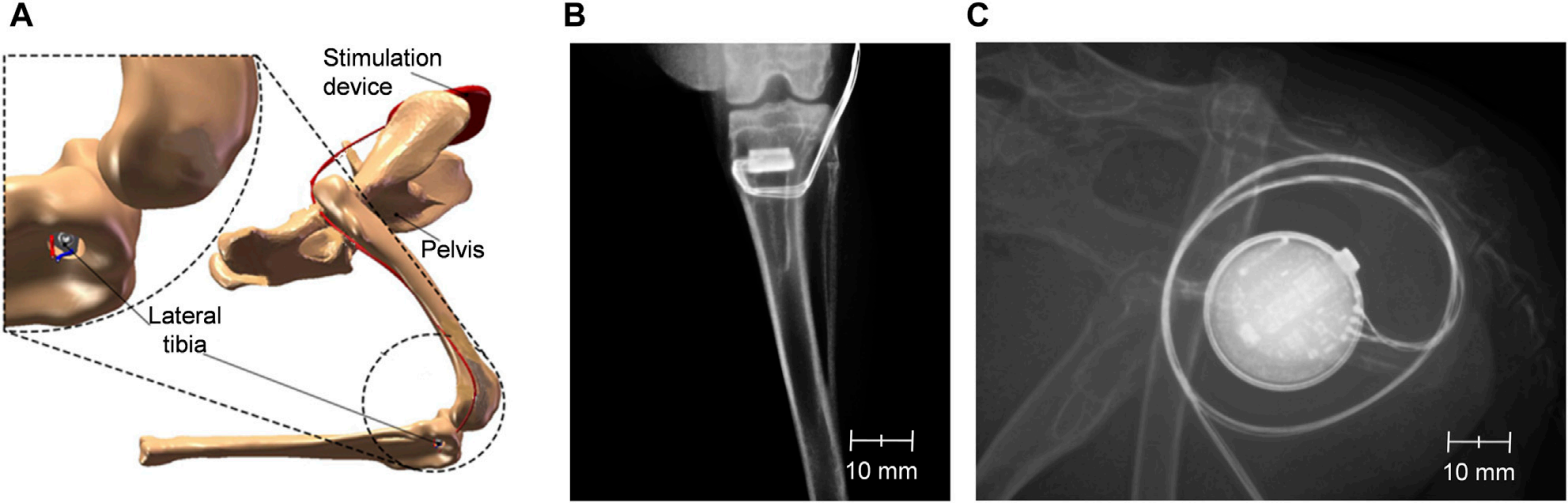
Positioning of the implants and the stimulation device in the hind legs of New Zealand white rabbits. Schematic depiction of the implants in the boreholes in the proximal tibia as well as of the stimulation device in the proximity of the pelvis **(A)**. X-rays of the hind legs and lower spine of a rabbit immediately after implantation showing a tibial implant **(B)** and a stimulation device with cables for two implants **(C)**.

Animals underwent the surgical procedure under general anesthesia, including ketamine (50 mg/kg of body weight, intramuscular; bela-pharmGmbH & Co. KG, Vechta, Germany) and xylazine (5 mg/kg of body weight, intramuscular; Bayer AG, Leverkusen, Germany). After general anesthesia was allowed to work for 10 min, metamizole at 40 mg/kg body weight and enrofloxacin (Baytril^®^ 40 mg/kg of body weight, Bayer AG, Leverkusen, Germany) were injected into the gluteal muscle. Both hind legs were then shaved and disinfected before the surgical site was numbed by subcutaneous administration of lidocaine hydrochloride (Xylocitin^®^ 2%; MIBE GmbH, Brehna, Germany). Stab incisions were made medially to the proximal tibial metaphysis to facilitate the positioning of the drill. Using a circular drill (diameter: 4.3 mm), bone defects of 8 mm length were prepared in the proximal tibia. Additionally, a lateral incision in the area of the proximal femur was used to form a skin pocket in the lateral pelvic region, and the miniature stimulation device was slid into the pocket (Figure 2). The implants were advanced under the skin towards the knee joint and inserted into the tibial defects by press-fit while taking care to avoid tensile stress on the connecting cables. The wounds were sutured with absorbable suture material and sealed with silver spray. The total duration of the surgery was 50–60 min.

In the first 5 days after surgery, the animals received subcutaneous injections of carprofen (5 mg/kg of body weight) and enrofloxacin (Baytril^®^ 40 mg/kg of body weight, Bayer AG, Leverkusen, Germany) and were supplemented with metamizole and a mixture of vitamins via the drinking water. All animals were weighed once a day and examined twice daily for signs of pain and wound-healing disorders. The first operated animal had to be euthanized early due to an implant infection. As a consequence, the sealing of the stimulation device was improved, and no further peri-implant infections were observed in the subsequently operated animals.

In these animals, electrical stimulation via the implants was started on the third postoperative day. Of the implants, one was electrically stimulated, while the other remained unstimulated and served as a negative control. The stimulated implants in the animals were subject to a stimulation protocol at 20 Hz of 3 × 45 min with 150 mV peak voltage daily, evenly distributed over a period of 12 h during daytime. Postoperatively and after 6 weeks, the animals were X-rayed to confirm the correct location of the implants and the stimulation device. Electrical stimulation was carried out for a total of 12 weeks, whereupon the animals were sacrificed. Subsequently, the tibiae were carefully dissected, freed of soft tissue, and fixed in a buffered formalin solution (4%). The osseointegration of the implant was evaluated by determining the bone-to-implant contact (BIC) with μCT and histomorphometry.

### 2.4 Histomorphometric analysis

The histomorphometric analysis was performed as previously described by Gabler et al. (2015). Briefly, the fixed bone specimens, including the implant, were dehydrated in a graded series of alcohol and embedded in polymethylmethacrylate before being cut with a cutting/grinding system with 0.1 mm diamond bands according to the thin-section technique by Donath and Breuner (Donath and Breuner, 1982). For each implant, one slice parallel to the base was prepared from the middle of the implant at exactly 3.5 mm from the edge. Subsequently, a microgrinding system (Exact 400CS; Exakt Apparatebau, Norderstedt, Germany) was used to reduce sections to a thickness of approximately 20 μm using 1200-grit sandpaper and to polish these afterwards with 4000-grit polishing paper. The bone sections were then stained with toluidine blue.

For histological evaluation, images were taken at 42.3× magnification under a digital microscope (VHX-6000, Keyence Deutschland GmbH, Neu-Isenburg, Germany). The histomorphometric images were analyzed with ImageJ (Schneider et al., 2012).

Bone-to-implant contact (BIC) in histomorphometry was quantified as the total length in micrometers (BIC length) that resulted from the sum of all segments with direct contact between toluidine blue stained bone and the implant surface. To calculate the percentage of BIC in relation to the total circumferential length of the implant, a polygonal frame was drawn around the implant corresponding to the maximum circumference. The recesses for the electrodes were subtracted from the length of the frame to determine the maximum length available for BIC. The percentage was calculated as the actual total length divided by the maximum available length of BIC.

**FIGURE 3:**
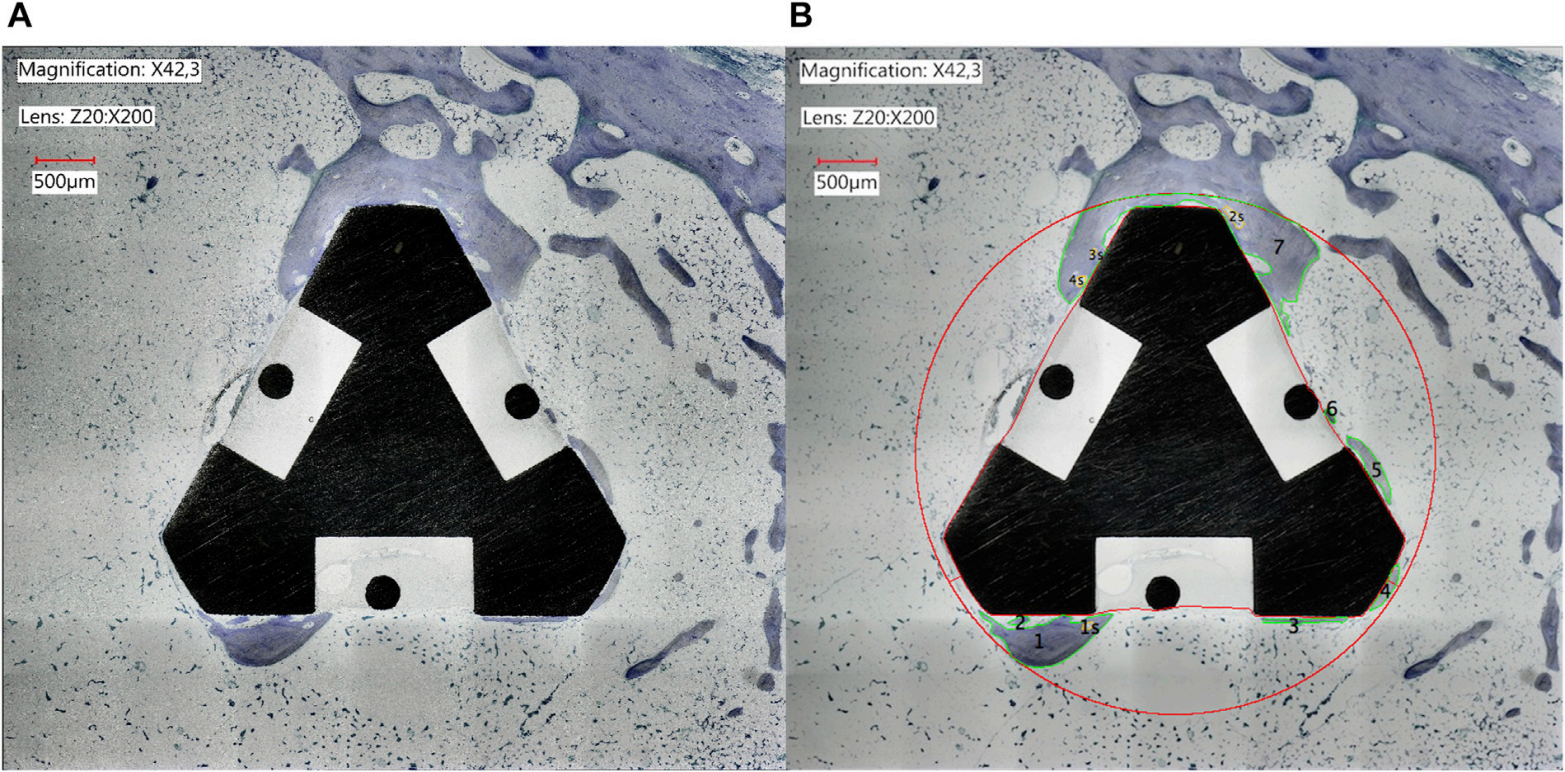
Histomorphometric image of the implant in the proximal tibia of a rabbit after osseointegration for 84 days. The bone and implant were sliced at 3.5 mm parallel to the base of the implant and stained with toluidine blue. **(A)** Original light microscopic image of a representative slice **(B)** Analysis of the image with ImageJ. After exclusion of the implant itself the area inside the red circle comprised the total analyzed area, while the green-rimmed areas represented the newly formed bone within the total area.

Additionally, the area of newly formed bone in the proximity of the implant was assessed by drawing a circle around the implant with a distance of 100 µm to each of the three corners of the triangular base and measuring the area of toluidine blue stained bone (Figure 3). The thus drawn circle covered a slightly bigger area than the initial borehole. The area of the implant was subtracted from the total area of the circle to calculate the percentage of newly formed bone in the proximity of the implant by dividing the blue stained areas by the total area of surrounding tissue in the circle.

### 2.5 μCT analysis

The acquisition of μCT image data of the specimens was performed with a microcomputer-tomograph Nanotom 180 nF (Phoenix nanotom, GE Measurement and Control solutions, phoenix|X-ray, Wunstorf, Germany). Voltage and current were set to 70 kV and 135 μA to reach the optimum contrast. The used μCT was a cone-beam computer tomograph with vertical specimen alignment. The samples rotated 360° in 0.75 steps. At each step, three 2D images were recorded; altogether, 1,440 2D images were acquired per sample. The reconstruction of CT data (composing X-ray 2D images to a 3D volume) was performed with the software datos|X-reconstruction (GE, Wunstorf, Germany). A beam hardening correction of 6.7 was used to compensate for the inhomogeneous reconstructed volume of the implant since titanium implants are harder to penetrate by X-rays than bone.

For further processing, the transformation of the volume data in the DICOM data was required. Each DICOM dataset had a maximum voxel edge resolution of 4–5 μm. The segmentation algorithm was executed on a workstation (Intel Quad Core Q9400 2.66 GHz, 2 GB RAM) with the segmentation software Amira 5.4.1 (FEI Visualization Sciences Group, Hillsboro, OR, United States) and consisted of the following processing steps: image data preprocessing, segmentation, and surface postprocessing. The segmentation algorithm comprised threshold detection of the histogram-oriented greyscale intensity of voxels, followed by inspection and interactive editing of the segmented areas in each slice of a dataset.

**FIGURE 4:**
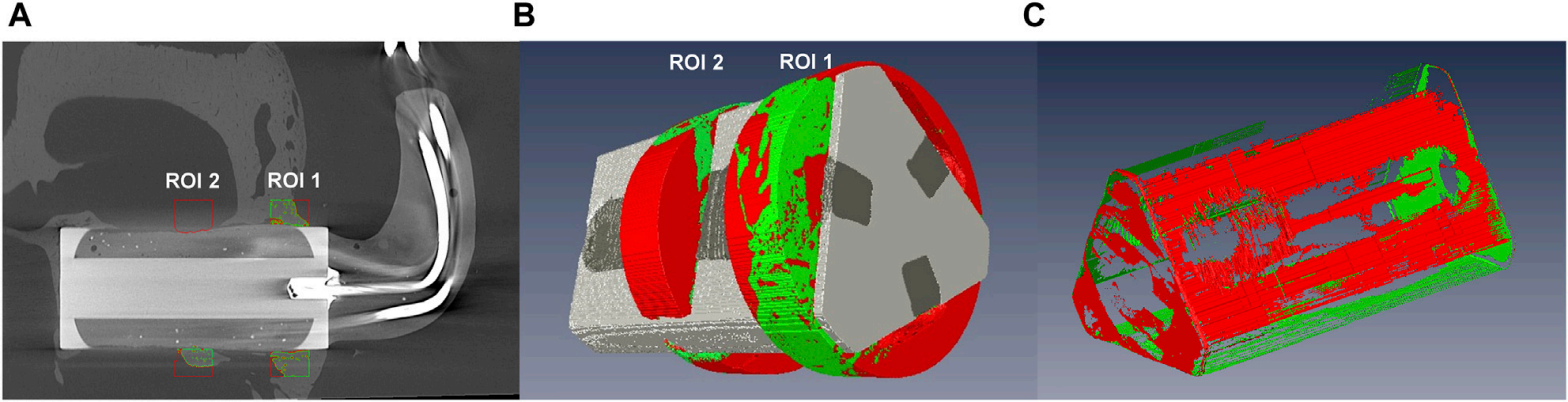
3D analysis with micro-computed tomography. **(A)** Image of an implant *in situ* with the red- and green-rimmed areas representing ROI 1 and ROI 2. **(B)** Image of a voxel-aided calculation of the bone volume in ROI 1 and ROI 2. Green regions represent newly formed bone within the defined space. **(C)** Image of a voxel-aided calculation of BIC area for the entire implant. Green areas represent areas with direct BIC.

Two regions of interest (ROIs) for the analysis of the reconstructed three-dimensional images were set within the range of the implant surface with approximately 1 mm surrounding bone tissue, as shown in Figure 4A. The first ROI was placed at 1 mm from the bony side of the implantation and was also 1 mm wide. ROI 1 anatomically indicated the cortical bone. The second ROI was located 3.5 mm from the edge of the implant and anatomically corresponded to cancellous bone and bone marrow. The starting points for both ROI were determined by selecting the sectional images in which the implant was visible, taking into account the Z-value, i.e., the sectional thickness of the image. Based on this information, the starting point of the ROI 1 was calculated as follows:

1 mmThickness of slice in mm=Number of the image at 1 mm from the edge

For each ROI, a circular area was placed around the implant base surface, which was then extended to a width of 1 mm to create a cylinder (3D) around the implant (Figure 4B). The bone volume was determined using the homogeneous Hounsfield unit (HU) of the bone. Within the ROIs, the titanium implant was marked and excluded from the calculation of the possible bone volume. The plastic coating of the electrode could only be segmented from the surrounding bone tissue by manual identification and subsequent correction, as automatic identification failed due to similar absorption values. Following this, the segmentation surface models were computed with a voxel-sustaining algorithm matching the exact voxel boundaries of each segmented material. Finally, the marked bone volume in mm^3^ was calculated using the Amira 5.4.1 software.

### 2.6 Statistical analysis

Data analysis and illustration were performed by GraphPad PRISM v.7.02 (GraphPad Inc., San Diego, CA, United States). Results are shown as box plots. Boxes depict interquartile ranges, horizontal lines within boxes depict medians, and whiskers depict maximum and minimum values. Data were analyzed regarding normal distribution with the Shapiro-Wilk test. Comparisons of original as well as percentage data of BIC length and the area of newly formed bone from the histomorphometric analyses (2D) between electrically stimulated and unstimulated samples were performed by t-test or Mann-Whitney test depending on the normal distribution of data. These tests were also used to compare µCT data (3D) of stimulated to unstimulated tibia samples. The analyzed µCT data included the BIC area and the bone volume in the cylinder around the entire implant (Figure 4C) as well as in the cylinders defined as ROI1 and ROI2. Since µCT data were scored blinded by two independent researchers, each score was incorporated as a single value in the statistical analysis. Significance was set to *p*-values less than 0.05. Further details of statistical tests are indicated in the results section and the figure legends.

## 3 Results

### 3.1 Electric field distribution in the animal bone based on numerical simulation

**FIGURE 5:**
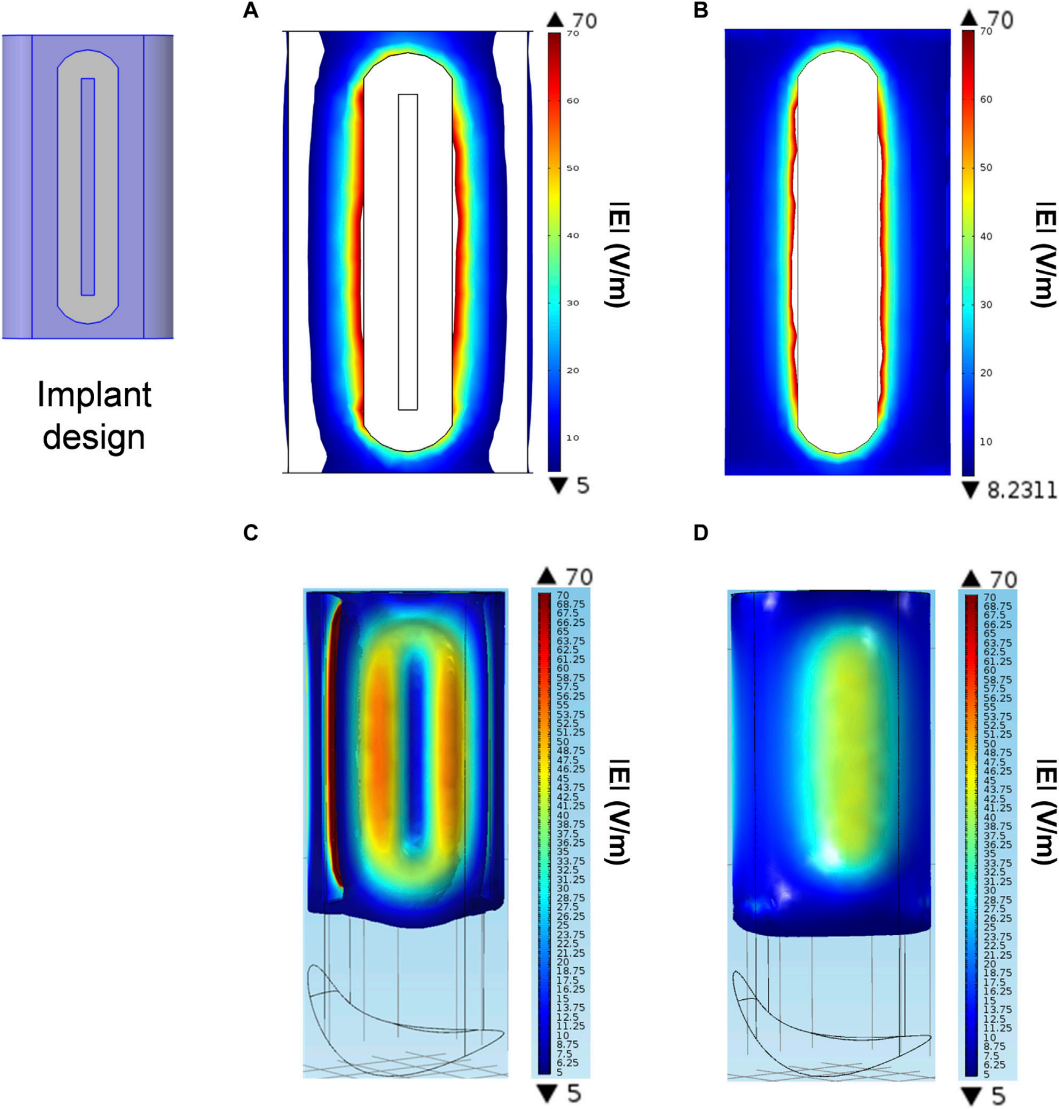
Numerical simulation of electric field distribution for the specific implant design in a gap model. **(A, B)** Electric field distribution on the implants’ electrode surfaces when gaps are either blood **(A)** or cancellous bone **(B)**. **(C, D)** Electric field distribution in the gaps between bone and implant when gaps are either blood **(C)** or cancellous bone **(D)**. The color-coded scales show the electric field strength in V/m and indicate the minimum (▼) and maximum (▲) values.

The parametric study, that was conducted within the range of 50–300 mV, showed that 150 mV peak voltage had to be applied to this particular implant design to achieve the optimal electric field of 5–70 V/m on the surface of the implants. This particular electric field strength resulted in the optimum effect on bone growth (Kraus, 1984). The electric field distribution around the implant in the hind limbs of the animals was calculated in the early phase of osseointegration when it is thought that the defect in the bone is filled with blood (Figures 5A,C) as well as in the later phase when the blood (or bone marrow) in the defect is replaced by newly formed bone (Figures 5B,D). Numerical simulation showed that not only on the implant´s surface, but also within the adjacent bone tissue the strength of the electric field ranged from 5 to 70 V/m. When gaps between bone and implant were considered as blood, the electric field on the surface of the chosen implant covered almost the entire implant electrode, except the wire electrode and the round edges of the implant. Due to its direct contact with the adjacent bone tissue, the round edge is of less importance in the defect model. When gaps were considered as cancellous bone, the electric field was distributed equally on nearly the entire surface of the implant electrode rather than the surface of the wire electrode.

The iso-surfaces of the electric field distribution in the gaps between bone and implant considering gaps as blood or cancellous bone are depicted in Figures 5C,D, respectively. In both cases the electric field within the stimulated tissue was homogenously distributed throughout the gaps around the implant.

### 3.2 Suitability of the rabbit model for the analysis of electrically stimulating bone implants

During the 84 days of stimulation, it became apparent that some of the electric circuits were unresponsive to the transmission of stimulation parameters by the radio transceiver. The days when a certain electrode stopped working and was no longer visible in the transmission software were recorded to determine the actual duration of electrical stimulation. Explantation of the stimulation devices after the sacrifice of the animals revealed that the failure was caused by a complete discharge of the battery. This was probably due to insufficient leak-proof sealing of the silicon sheathing. In total, two implants were electrically stimulated over the entire duration of 84 days, while three further implants that were stimulated for at least 3 weeks stopped working after 19, 31, and 43 days, respectively. All implants, including the five unstimulated control implants, remained in the rabbit knees for the entire period of 84 days.

### 3.3 Analysis of bone-implant contact in histomorphometric images

**FIGURE 6:**
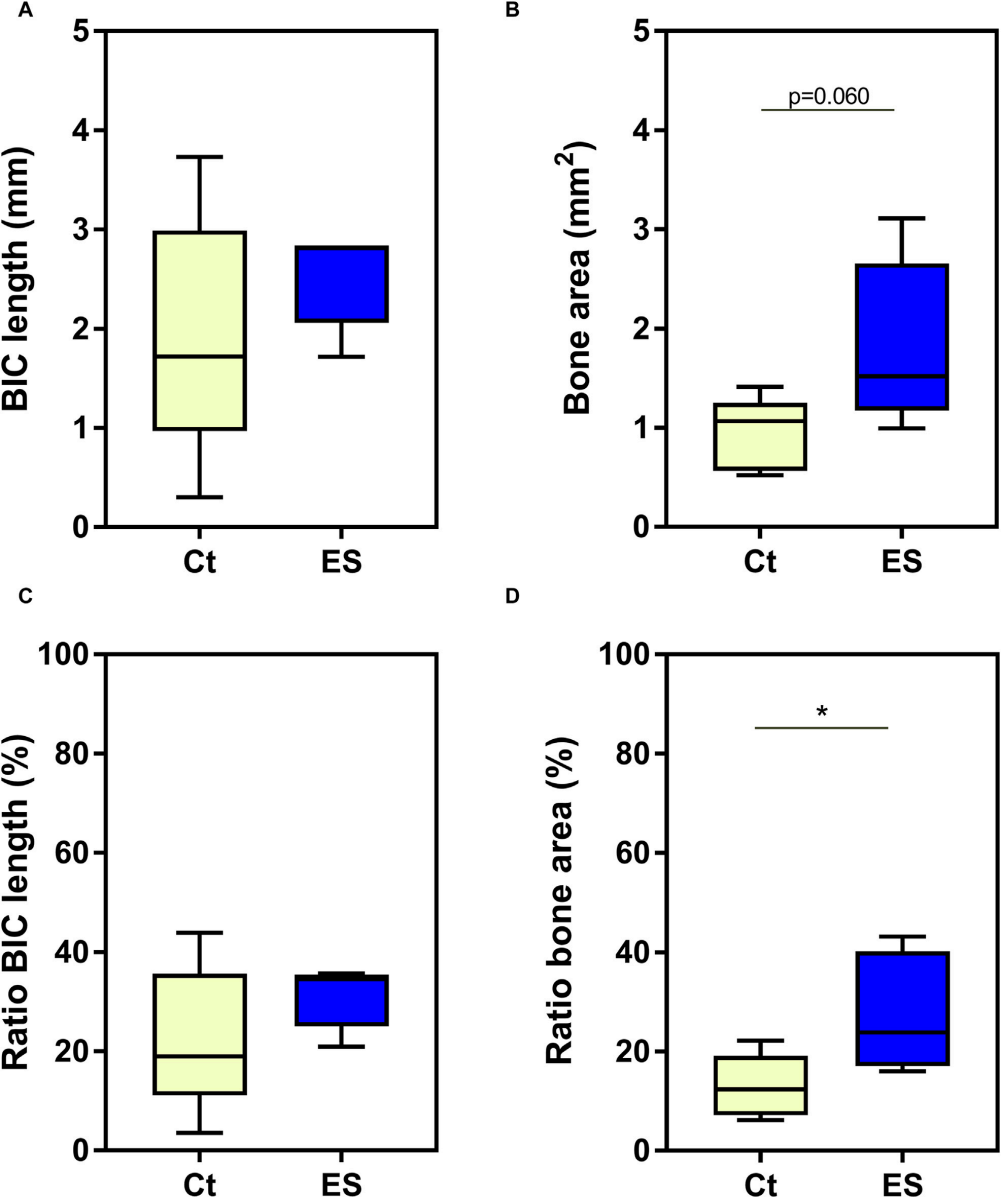
Histomorphometric 2D Analysis. Absolute data **(A, B)** and ratios **(C, D)** of the length of bone implant contact (BIC length) **(A, C)** and area of newly formed bone (bone area) **(B, D)**. Ct = unstimulated control; ES = implants stimulated with 150 mV, thrice daily for 45 min. Statistical analysis was performed with unpaired t-test for normally distributed values, while Mann-Whitney test was used, when data were not normally distributed. Trends or significant differences between Ct and ES are indicated: *p < 0.05.

In the histomorphometric images, the length of BIC (mm) as well as the area of newly formed bone (mm^2^) within the bone defect created by the borehole was determined for tibia samples stimulated with 150 mV (3 × 45 min/day) and the unstimulated controls. When comparing the five stimulated and five unstimulated samples, there was no effect of stimulation on the length of BIC (*p* = 0.3095, Mann-Whitney test, Figure 6A), but a trend for an increase in bone area in the defect after electrical stimulation (*p* = 0.0600, unpaired t-test, Figure 6B). However, since the cut for the image slice was not always set exactly parallel to the base of the implant, there were distortions regarding the length and area between the images. To address this bias, the ratios of actual length and area to possible length and area as determined in the respective image were calculated and expressed as percentages. The comparison of the percentage values showed a significant effect of electrical stimulation on the area of newly formed bone (*p* = 0.0418, unpaired t-test, Figure 6D). On average, 27.7% of the defect area was filled with newly formed bone after thrice daily stimulation for 45 min with 150 mV, while without electrical stimulation only 13.0% of the defect area represented new bone. However, there was no influence on ratios of BIC length (*p* = 0.2629, unpaired t-test, Figure 6C).

### 3.4 Analysis of bone-implant contact in micro-computed tomography (3D)

**FIGURE 7:**
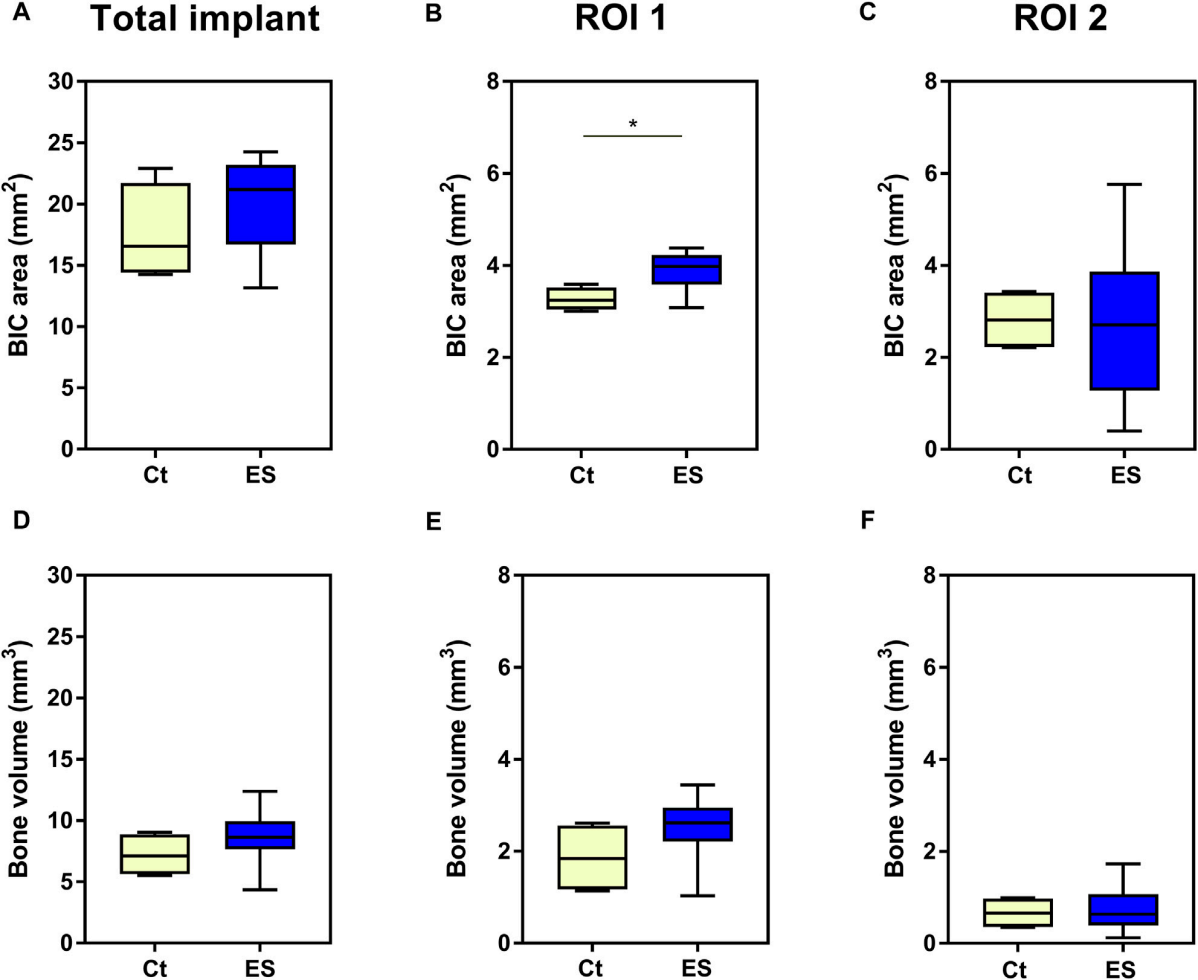
3D analysis of osseointegration. Comparison of unstimulated control (Ct) and electrical stimulated implants (ES) for area of bone implant contact (BIC area) **(A–C)** as well as newly formed bone volume **(D–F)** determined for the entire implant **(A, D)**, in region of interest (ROI) 1 **(B, E)** and ROI 2 **(C, F)**. Statistical analysis was performed with unpaired t-test for normally distributed values, while Mann-Whitney test was used, when data were not normally distributed. Significant differences between Ct and ES are indicated: *p < 0.05.

The results of the µCT analyses are depicted in Figure 7. When analyzing the BIC area for the entire implant as well as the volume of newly formed bone in the entire defect space, there were no significant effects of electrical stimulation despite the observation of higher mean values for both measures after stimulation with 150 mV compared to unstimulated control (BIC area: 20.2 mm^2^ vs. 17.6 mm^2^; Bone volume: 8.6 mm^3^ vs. 7.2 mm^3^ for thrice daily stimulation for 45 min with 150 mV vs. unstimulated control, respectively). The detailed analysis of the two different ROIs showed a significant increase of BIC area in the region of cortical bone (ROI 1, *p* = 0.0190, unpaired t-test, Figure 7B) but not in the cancellous bone or intramedullary (ROI 2). No differences were observed in bone volume in the ROIs (Figures 7E,F). It is interesting to note that there were significant differences in the volume of newly formed bone between the two ROIs, with higher bone volume in the cortical region compared to the cancellous region. This was observed for the electrically stimulated (*p* < 0.0001, paired t-test) as well as unstimulated bone samples (*p* = 0.0112, paired t-test).

## 4 Discussion

In this study, the effects of electrical stimulation with 150 mV three times per day for 45 min on the osseointegration of a titanium implant in a defect model in the proximal tibia of New Zealand White rabbits were analyzed. The stimulation parameters of three times per day for 45 min were based on the recommendation for electrical stimulation with the Magnetodyn^®^ system (Neue Magnetodyn GmbH, Munich, Germany), also temporarily distributed as the ASNIS™ III s-series screw (Stryker, 2010), for the treatment of avascular necrosis of the femoral head (Ellenrieder et al., 2013; Hiemer et al., 2016; Ellenrieder et al., 2023). The numerical simulation of the electric field distribution showed that electrical stimulation with 150 mV peak voltage led to homogeneous fields in the vicinity of the implant with 5–70 V/m. These values result in reproducible bone growth *in vivo*, according to Kraus (Kraus, 1984). This was confirmed by the beneficial effect on implant osseointegration observed in our present pilot study. However, while there was an overall increase in BIC after electrical stimulation, the effects were only significant for the ratio of the area of newly formed bone in the 2D analysis and the BIC area in the 3D analysis in ROI 1. Whereas the percentage of newly formed bone in the 2D analysis more than doubled after electrical stimulation, the changes in the 3D analysis were not so pronounced, with increases of 15%–30% after stimulation in nearly all the measured parameters. The absence of an effect in the medullary cavity suggests that while electrical stimulation induces bone formation, it does not lead to calcification in these regions.

There are only a few studies in various animals, such as rabbits, dogs, and sheep, that used electrical stimulation with direct coupling for implant osseointegration (Park et al., 1980; Buch et al., 1984; Narkhede, 1998; Shayesteh et al., 2007; Dergin et al., 2013; Bins-Ely et al., 2017; Zhou et al., 2019; Dingle et al., 2020). In the early studies, the electrodes were placed in the vicinity of porous implants (Park et al., 1980) or titanium implants which contained small canals (Buch et al., 1984). Both animal studies were designed to observe the ingrowth of bone into the pores or the canals. The results from the studies showed that electrical stimulation with 8 µA or 5 µA and 20 µA led to higher interfacial shear strength of bone and implant in a tensile test (Park et al., 1980) and increased average ingrowth into the canals by 50%–60% (Buch et al., 1984), respectively. In further animal studies, the metallic implant itself served as a cathode. In the study by Narkhede et al., the cathode, as represented by the titanium implant in the mandible of New Zealand White rabbits, and the anode on the ears of the animals were connected to an external power source (Narkhede, 1998). This design had the disadvantage that the animals had to be sedated each time the electric current was applied, thus clearly limiting the duration and the number of stimulation treatments. Bins-Ely et al. (Bins-Ely et al., 2017) circumvented this problem by directly incorporating the electric source into the titanium dental implants that were then implanted into the tibia of Beagle dogs. The cathode of the electric source was connected to the part of the implant inserted into the bone, while the anode was in contact with the soft tissue of the tissue flap that closed the incision (Bins-Ely et al., 2017). In this experimental design, electrical stimulation with a continuous direct current of 20 μA, but not with 10 μA, significantly increased BIC after 15 days of stimulation. However, this design did not allow to vary the duration or the frequency of the treatment.

Finding the optimum parameters for electrical stimulation to elicit the intended biological response without overstimulation is still an important issue since the wide variety of the reported dosages and regimens led to confusion and hampers the use of electrical stimulation as a treatment option (Bhavsar et al., 2020). The variation of frequency or duration of treatments was mainly investigated in animal studies, which used pulsed electromagnetic fields (PEMF) to improve implant osseointegration. In an animal study with male Wistar rats, PEMF stimulation with 1 hour per day showed better results than stimulation with 3 hours per day in removal torque tests, bone volume and bone mineral density, while trabecular bone thickness and early osseointegration were higher after electrical stimulation with 3 hours per day (Nunes et al., 2021). Matsumoto et al. (2000) also concluded that apart from determining the minimal necessary intensity of electrical stimulation, the duration of treatment per day as well as the total period of electrical stimulation influenced the outcome regarding osseointegration. In their animal study with Japanese white rabbits there was a significant increase in BIC ratio in the femurs with rough surface Ti6Al4V implants after PEMF for periods of one and 2 weeks and for bone area ratio after 2 weeks compared to the control group, but after 4 weeks no more differences were apparent.

While Buzzá et al. (2003) recorded an increase of newly formed bone and higher removal torque after 6 weeks compared to 3 weeks after implantation of titanium dental implants into the tibial plateaus of white rabbits, there were no significant differences between electrical stimulation and control at these late time points. This leveling effect was not only shown in small animal models but also in large animals like sheep. At a post-implantation period of 4 weeks, mature bone contact to titanium dental implants in the tibia of sheep was established after DC stimulation but was not observed in the control group, thus supporting the osteogenic effect of the electrical stimulation. However, at a later time point of 8 weeks the electrical stimulation as well as the control group showed both mature bone contact of the implants without significant differences between the groups (Dergin et al., 2013). Taken together, the results from the animal studies suggest that electrical stimulation initially accelerates osseointegration, however, at later time points “normal” osseointegration equalizes the advantage in healthy animals (Barak et al., 2016; Pettersen et al., 2022). This would also explain why the benefits that we observed after up to 12 weeks of electrical stimulation in this study were only on a small scale and often not significant for the determined measures. However, when bone metabolism and regeneration are impaired, the advantage offered by electrical stimulation might counteract negative outcomes and clearly benefit the osseointegration of implants. Indeed, in disease models of diabetic and osteoporotic rabbits, osseointegration of porous titanium scaffolds was still significantly better in the PEMF-treated group after 8 and 12 weeks, respectively, than in the diseased control group (Cai et al., 2018; Ye et al., 2021). In the diabetic animals, PEMF treatment could completely alleviate the detrimental effects of type 1 diabetes mellitus on bone metabolism, and at 8 weeks, diabetic rabbits with electrical stimulation showed the same level of osseointegration as healthy control animals (Cai et al., 2018).

Apart from a direct effect on bone remodeling, electrical stimulation might also suppress inflammatory processes that lead to bone erosion, as shown in a mouse model of rheumatoid arthritis (Hong et al., 2023). But it is likely that these additional biological effects also depend on frequency, amplitude, timing, and length of exposure. The discussed animal studies show that PEMF stimulation is suitable to elucidate the optimal exposure parameters, however, its use is limited by the need to restrain the animal during the treatment. The presented animal model allows the testing of different frequencies, timings, and periods, but – most importantly – by using an implanted stimulation device with a radio transceiver, the animals can move freely in their cages postoperatively and do not have to be restrained or sedated during electrical stimulation. Additionally, the radio transceiver also provided feedback on whether the stimulator was working properly. Therefore, in combination with the use of an alternating electric field and the thus resulting advantages, the animal model in this pilot study is unique for the investigation of implant osseointegration. To our knowledge, there is only one other animal study in dogs that used a biphasic electrical current to stimulate bone formation around an implant (Song et al., 2009). A biphasic or alternating electrical current offers the benefit that there are no changes in pH in the tissue surrounding the electrodes and no bone erosion associated with the anode (Thrivikraman et al., 2018; He et al., 2022). In contrast to our study, the stimulation parameters in the canine study were set in advance and stored on the microchip that was located inside the implant and also contained the oscillator to generate the biphasic electric current. Thus, stimulation parameters were fixed at a current amplitude of 20 mA/cm^2^, a duration of 125 ms, and a pulse rate of 100 pulses/s for continuous stimulation. Under these conditions, BIC was only significantly higher at week 3 with 165%, but there were no significant differences between electrically stimulated implants and controls at week 5. However, the formation of new bone area was 1.3 and 1.35 times higher after electrical stimulation at three as well as 5 weeks, respectively. The observed results in the study of Song et al. were very similar to the here presented data (Song et al., 2009).

In our present pilot study, it was confirmed that the developed experimental design with an electrical stimulation system, which is directly located in the tibia of rabbits, allows variations in the stimulation parameters throughout the experiment and provides feedback regarding the integrity of the stimulation device. Electrical stimulation significantly enhanced osseointegration despite the fact that the time point of analysis was relatively late at 12 weeks post implantation, a period when osseointegration might be complete in healthy animals without additional electrical stimulation (Pandey et al., 2022). Therefore, at an earlier time point or in disease models, the developed implant seems to be well suited for future investigations of optimal stimulation parameters in electrical stimulation. However, there are some limitations to the system used. As the miniature stimulation device was still too large to fit into the implant itself, the spatial arrangement required the use of cables. Due to the unimpeded movement of the animals, these cables are prone to be torn despite the extra care to avoid tensile stress on the connecting cables during implantation. It would, therefore, be preferable to integrate the stimulation device directly into the implant to avoid the use of the connecting cables. While further attempts were made to miniaturize the stimulation device for the use in small animal models (Plocksties et al., 2021), this system still uses wires. However, the device might be incorporated in the implant or joint endoprostheses of larger animals. Furthermore, the replacement of the battery on the device by implantable energy harvesters (IEHs), which use piezoelectric effects, could reduce the problems associated with battery breakage, dislocation, and exchange (Liu et al., 2021). Regarding these new developments, the use of direct coupling of electrical stimulation by an alternating field might provide a valuable treatment option to aid the osseointegration of permanent bone implants, especially in patients with impaired bone metabolism. Despite the use of implant surfaces that facilitate osseointegration, certain comorbidities such as osteoporosis and inflammatory conditions still represent significant risk factors for implant loosening, probably due to delayed osseointegration (Aro et al., 2012; van Hamersveld et al., 2021; He et al., 2024). Accelerating osseointegration in those patients by using an electrically stimulating implant might prevent loosening and enhance the survival of uncemented joint endoprostheses. However, the clinical application would require to define the dose-response curve and the optimal duration of the stimulation treatment, in particular in relevant disease models.

## Data Availability

The raw data supporting the conclusions of this article will be made available by the authors, without undue reservation.
